# Can traits predict individual growth performance? A test in a hyperdiverse tropical forest

**DOI:** 10.1111/nph.15206

**Published:** 2018-05-18

**Authors:** Lourens Poorter, Carolina V. Castilho, Juliana Schietti, Rafael S. Oliveira, Flávia R. C. Costa

**Affiliations:** ^1^ Forest Ecology and Forest Management Group Wageningen University and Research PO Box 47 AA Wageningen 6700 Netherlands; ^2^ Coordenação de Pesquisa em Biodiversidade Instituto Nacional de Pesquisas da Amazônia (INPA) Caixa Postal 2223, CEP 69008‐971 Manaus Brazil; ^3^ Embrapa Roraima Rodovia BR 174, Km 8, Distrito Industrial, Caixa Postal 133, CEP 69301‐970 Boa Vista RR Brazil; ^4^ Depto. de Biologia Vegetal Instituto de Biologia Universidade Estadual de Campinas Caixa Postal 6109, CEP 13083‐970 Campinas SP Brazil

**Keywords:** acclimation, Amazon, defense, functional traits, growth, plant strategies, plasticity, tropical rainforest

## Abstract

- The functional trait approach has, as a central tenet, that plant traits are functional and shape individual performance, but this has rarely been tested in the field. Here, we tested the individual‐based trait approach in a hyperdiverse Amazonian tropical rainforest and evaluated intraspecific variation in trait values, plant strategies at the individual level, and whether traits are functional and predict individual performance.
- We evaluated > 1300 tree saplings belonging to > 383 species, measured 25 traits related to growth and defense, and evaluated the effects of environmental conditions, plant size, and traits on stem growth.
- A total of 44% of the trait variation was observed within species, indicating a strong potential for acclimation. Individuals showed two strategy spectra, related to tissue toughness and organ size vs leaf display. In this nutrient‐ and light‐limited forest, traits measured at the individual level were surprisingly poor predictors of individual growth performance because of convergence of traits and growth rates.
- Functional trait approaches based on individuals or species are conceptually fundamentally different: the species‐based approach focuses on the *potential* and the individual‐based approach on the *realized* traits and growth rates. Counterintuitively, the individual approach leads to a poor prediction of individual performance, although it provides a more realistic view on community dynamics.

## Introduction

Over the past two decades, the field of functional ecology has been booming and blooming. Functional ecology has such a wide appeal because it can provide a mechanistic understanding of how traits affect plant performance, and hence community assembly and dynamics (Grime *et al*., 1997; Westoby, 1998). Functional ecology, therefore, has the potential to turn ecology from an often descriptive into a more mechanistic and predictive science (McGill *et al*., 2006). This promise has led to a surge of comparative studies in which traits and trait spectra, such as the leaf and stem economics spectrum, have been measured, assuming that these traits are functional and have an impact on plant performance (Wright *et al*., 2004; Baraloto *et al*., 2010). Many controlled experiments have indeed shown that a suite of traits is closely associated with plant growth (e.g. Lambers & Poorter, 1992) and survival (Kitajima, 1994). The question is, however, whether these relationships are ecologically relevant and still hold under natural conditions.

Field studies assessing the relationship between traits and demographic rates have mostly focused on trees, because they can easily be monitored for their diameter growth and survival. These studies have shown that, across species, trait values that enhance resource acquisition and resource use efficiency (such as high specific leaf area (SLA) and low wood density (WD)) are associated with fast growth, whereas trait values that enhance resource conservation (low SLA and high WD) are associated with high survival (Poorter & Bongers, 2006; Poorter *et al*., 2008; Martínez‐Vilalta *et al*., 2010; Rüger *et al*., 2012).

Most of these comparative studies have focused on species, and related average trait values to average species performance, thus ignoring intraspecific trait variation and environmental variation. This is somewhat surprising, as a functional trait is defined as any morphological, phenological, or physiological features of an *individual* that affect the growth, survival, reproduction, and fitness of that individual (Violle *et al*., 2007). Recently, there has been a plea to take this intraspecific trait variation explicitly into account (Albert *et al*., 2011). Plasticity is here defined as the ability of a species to vary in its trait values, and acclimation, as the phenotypic adjustment of a genotype to the environment, thus potentially enhancing its performance (Poorter *et al*., 2010). It should be said that not all trait variation implies environmental acclimation, as trait values may also vary within species, due to variation in plant size and ontogenetic plant development (Evans, 1972), variation in organ size (Milla & Reich, 2007), age (Kitajima *et al*., 2002), genetic variation, biotic interactions (Friesen, 2011), and tissue wear.

Given that most evaluations of the relationships between tree traits and performance have been done at the species level, a clear demonstration of the importance of traits to the fitness at the individual level is lacking. Here, we present data on environmental conditions, individually measured trait values, and growth performance of over 1300 tropical tree saplings from a hyperdiverse Amazonian tropical rainforest. The forest grows on highly weathered and extremely nutrient‐poor soils, and is characterized by tall and slender trees with narrow crowns that cast a deep shade with little light variation in the understory. We therefore evaluated 25 functional stem and leaf traits that are related to light capture, carbon and nutrient conservation, plant defense, and persistence. We address the following three questions.

First, how much trait variation is observed in the community, and how much of the trait variation is found within and across species? We hypothesize that size‐related traits (e.g. leaf area) are strongly related to tree size, and that light‐capture‐related traits (e.g. SLA and Chl content) are strongly related to light, and therefore vary mostly within species. We hypothesize that traits related to tissue quality (e.g. leaf dry matter content) and toughness are phylogenetically more conserved (Chave *et al*., 2006). The fact that traits are conserved suggests that they are less plastic, and therefore these conserved traits should show less trait variation within species.

Second, how are traits associated at the individual level, and does this parallel the interspecific relationships? We hypothesize that, across individuals, the same leaf and stem economics spectra are found as reported in the literature across species but that the strength of these relationships may be weaker because of the lower organizational scale considered (individual rather than species), and because we focus on a single community, where there is potentially less variation in environmental conditions and less variation in trait values (Messier *et al*., 2017). We nevertheless expect high SLA being associated with low leaf thickness and low leaf density (Villar *et al*., 2013) and high WD being associated with high bark density and high stem and bark dry matter content (Baraloto *et al*., 2010; Poorter *et al*., 2014).

Third, to what extent is plant performance shaped by the environment (light, water, and nutrients) and the attributes of the individual (size and traits)? We hypothesize that plant growth should increase with resource availability (light, water, soil nutrients), with plant size, and with traits that enhance leaf area accumulation, light capture, and resource conservation in the shaded understory (i.e. low SLA, high leaf density, Chl content and leaf area) (cf. Lusk, 2002).

## Materials and Methods

### Study site

The study was conducted at Reserva Florestal Adolpho Ducke, located 26 km north‐west of Manaus (02°55′S, 59°59′W at the reserve headquarters). The reserve covers 10 000 ha (10 km × 10 km) of terra‐firme tropical rain forest, with a closed canopy 30–37 m tall and emergent trees up to 40–45 m. Mean annual temperature is 26°C, and mean annual rainfall between 2400 and 2700 mm yr^−1^, with a dry season between July and October (Marques‐Filho *et al*., 1981). Altitude varies from 30 to 120 m, but local altitudinal differences between valleys and the nearest plateaus range from 10 to 30 m. Soils are derived from tertiary marine sediments from the Alter‐do‐Chão formation and represent a continuum from clayey latosols on the ridges to sandy podzols in valleys (Chauvel *et al*., 1987).

### Sampling design and measurements

We collected trait data for 1320 tree individuals distributed in 17 permanent plots covering a gradient of topographic conditions, from the high clayey plateaus to the sandy valleys. These plots are widely distributed across Reserva Ducke, at least 1 km distant from each other, and each plot follows an altitudinal contour, so within‐plots altitude, distance to the water table, and soil are constant. These individuals represent 383 named species (33% of the 1176 tree species found in the reserve), plus 267 unidentified individuals.

We sampled all tree saplings with diameter at breast height (DBH) between 1 and 5 cm in a strip of 250 m × 1 m per plot, which resulted in an average of 74 ± 22 individuals per plot. Traits were collected from October 2014 to January 2016, after the last plot census that ended in August 2014. We did not collect material for trees with only one branch, for which collection would probably induce death of the individual. For the remaining individuals, we measured total tree height with poles and tape, estimated crown exposure index according to Keeling & Phillips (2007) and collected a branch at least 40 cm long. For plants with only short branches, the longest ones were collected. Branches were taken from the most illuminated side of the crown. We tried to avoid visually unhealthy leaves and leaves with epiphylls, but this was not always possible, so we sampled the best from what was available.

We counted leaves for a 40 cm branch piece (or, if less, noted branch length for posterior standardization), took the best two leaves for fresh and dry weights and leaf area, took a 4–5 cm terminal piece of the branch for fresh and dry weights and volume, and a small 1 cm branch piece next to the first for macro‐anatomical measurements. Leaves were measured for thickness with a micrometer (middle part, avoiding main veins) and for Chl content with a SPAD meter (Minolta SPAD 502 Chlorophyll Meter, Spectrum Technologies Inc., Plainfield, IL, USA). The force to punch a leaf was measured using a purpose‐built penetrometer (Rozendaal *et al*., 2006) that measures the mass needed to punch the flat‐ended part of a nail through the leaf. Leaves were subsequently scanned for their area, weighed for fresh mass, and dried for 48 h at 60°C for dry weight. Petioles and rachises were weighed separately from the leaf lamina. Branch pieces were weighed, volume was determined with the water displacement method, both with and without bark, and dried at 105°C for 3–4 d for dry weights. Anatomical measurements of pith, xylem, and bark diameter of the branch were taken with a caliper under a stereo‐microscope.

Growth data were obtained from the tree measurements taken in 2007–2009 (census 3) and 2014 (census 4). The interval between censuses per plot varied between 5.3 and 7.2 yr. We calculated basal area growth rate as the difference between final and initial basal area, divided by the census interval.

Environmental data were taken at plot level for height above the nearest drainage (HAND) and soil fertility, and at the individual level for light (with the crown exposure index). HAND (in meters) is a descriptor of drainage potential, related to the distance to the water table (Rennó *et al*., 2008). Soil fertility was measured using the total exchangeable bases (in centimole equivalents per kg soil), obtained from a compound soil sample 5 cm deep, from six locations along the plot, and analyzed at the Soil Laboratory of the Agronomy Department at INPA.

### Trait calculations

Based on the initial measurements, we calculated the following traits for each individual. Branch cross‐sectional area (BA, cm^2^) was calculated as *π*(0.5 × branch diameter)^2^, and is an indicator of the investment in biomechanical and hydraulic support of the branch. Branch leaf area (BLA) was calculated as the number of leaves on the branch, multiplied by the average leaf area of the two leaves that were collected for the branch. Most branches had a length of 40 cm, but because some individuals had shorter branches, we standardized the BLA by dividing it by the branch length (BLA_bl_, cm^2^ cm^−1^). The BLA_bl_ indicates the light‐capture potential of the branch. Similarly, to account for differences in branch length, we calculated the leaf number per unit branch length (LN_bl_, cm^−1^), which indicates how efficiently branches pack their leaves and produce a higher number of lateral buds.

The density and dry matter content of wood, bark, and whole branch were calculated based on the small branch sample (*c*. 5 cm long). Wood density was calculated as branchwood dry mass over branch fresh volume (WD, g cm^−3^), bark density as bark dry mass over bark fresh volume (BD, g cm^−3^), branch density (g cm^−3^) as branch dry mass (including wood and bark) over branch fresh volume, and wood dry matter content (WDMC, mg g^−1^) and bark dry matter content (BDMC, mg g^−1^) as the dry mass divided by the fresh mass of that tissue. Specific branch length (SBL, cm g^−1^) was calculated as the length of the small branch sample divided by its dry mass, as is an indicator of the biomass efficiency for branch expansion.

Specific leaf area (SLA, m^2^ kg^−1^) is the leaf area per unit leaf dry mass. It was calculated by pooling the two leaves that were collected per branch, and by dividing their leaf area over their dry mass. Petioles were not included in the SLA calculation as they can be very large for rainforest species, and petioles are more related to leaf positioning than biomass efficiency for leaf display. For compound leaves, SLA was based on all the leaflets of the two leaves, and the rachis was not included in the SLA calculation. Leaf area ratio (LAR, cm^2^ g^−1^) at the branch level was calculated as the total leaf area of the branch divided by the whole dry biomass of the branch (i.e. the sum of the mass of the branch, leaves, and petioles). Total branch dry mass was calculated as the branch volume (cm^3^) multiplied by the branchwood density (g cm^−3^) of the branch sample. Branch volume was estimated based on branch diameter and branch length, assuming the shape of a paraboloid; 0.5 × BA × Branch length. Total leaf and total petiole mass were calculated by multiplying total leaf number by the leaf mass or by petiole mass of an individual leaf respectively. The leaf mass fraction (LMF, g g^−1^) at the branch level is the total leaf mass divided by the whole dry biomass of the branch (i.e. the sum of the mass of the branch, leaves, and petioles). LMF was calculated as LAR divided by SLA. LMF indicates how much of the biomass of the branch is allocated to leaves for light capture, SLA indicates the biomass efficiency of leaf display at the leaf level, and LAR indicates the same at the branch level. Chl content per unit leaf area (μg cm^−2^) was calculated based on the SPAD values using an equation for rainforest trees (Coste *et al*., 2010): Chl = (117.1 × SPAD)/(148.84 – SPAD). The Chl content indicates the light‐harvesting potential of the leaf.

The leaf dry matter content (LDMC, mg g^−1^) and petiole dry matter content (PDMC, mg g^−1^) were calculated as the dry mass over the fresh mass of these tissues, and indicate their toughness and material construction cost. Leaf density (LD, g cm^−3^) is the leaf dry mass per unit leaf volume, and it was calculated as 1/(SLA × leaf thickness). High leaf density implies structural enforcement of the leaf, but it also leads to slow internal CO_2_ diffusion and, hence, to slow assimilation rates (Niinemets, 2001). The force to punch (FP, N cm^−1^) is the total force needed to fracture a length of leaf. It was calculated as the mass (in grams) that was needed to punch the leaf (the sum of the mass of the water, recipient, syringe, and nail) multiplied by 0.0098, divided by the circumference of the nail head (1.1 cm). The specific FP (FPs, N cm^−2^) is the force needed to facture a cross‐sectional area of leaf. It was calculated as the FP divided by the leaf thickness and indicates the material strength of the leaf tissue.

### Analyses

To evaluate how much of the trait variation is observed within species, we selected 29 species that had at least five individuals (median 7, range 5–40). We used five individuals as a threshold, because this number is often recommended (Pérez‐Harguindeguy *et al*., 2013) as the minimum required to quantify species‐specific trait values. For each trait we did an ANOVA with species as factor and calculated the variance explained by species. By definition, the remainder of the trait variance is then due to within‐species variation or measurement error.

To evaluate how traits were associated in multivariate plant strategies, we did a principal components analysis (PCA) using the 25 traits and 930 of the 1320 individuals that did not have any missing trait values. Pairwise trait relationships were subsequently analyzed with Pearson correlations and regression analyses.

To evaluate to what extent plant size, traits, and environment could explain individual tree growth, we fit a series of nine mixed models, in which basal area growth rate was the response variable, the 17 plots were included as a random effect, and plant size (in most models plant height, in one model plant height and basal area), traits, and environmental conditions (the light conditions of the individuals, and the HAND score and soil cation concentration of the plot) as continuous variables. We selected six traits that were associated with the strategy spectra that we distinguished in the PCA. We used WD and FP as indicators of the axis of tissue toughness, leaf size, branch area, and the proportion of cross‐sectional branch area in xylem as indicators of organ size, and SLA as indicator of efficient leaf display. We ran nine models that had an increasing complexity: from only size and environment, or size and traits, to a complete set of size, traits, and environment, and complementary models in which only the best environment and best traits (those with significant effects in previous models) were included, always together with size. Before analysis, all continuous variables were standardized, whereby each cell is subtracted from the variable mean and then divided by its standard deviation. This allows comparison of the standardized regression coefficients and effect sizes of the independent variables. Because trait–growth rate relationships may vary with topographic position or light conditions, we also included models with a trait–HAND interaction and a trait–light interaction. Models were ranked based on the Akaike information criterion values. All models were run with package lme4 (Bates *et al*., 2014) and *R* ^2^ for fixed and random factors calculated with MuMin (Bartoń, 2013), in the R statistical environment (R Core Team, 2014).

To evaluate whether a species‐based approach shows different relationships between traits and growth rates than an individual‐based approach, we selected 36 species that had at least five individuals, providing a total of 370 saplings. Correlations between traits and growth rate were then evaluated using the individual‐based approach (using the 370 individuals as replicates) and the species‐based approach (by averaging traits and growth rates per species and using the 36 species as replicates).

## Results

### Trait variation

Trait values varied substantially across saplings within this tropical community. For example, when the 5^th^ and the 95^th^ percentiles of the trait values are compared, WD varied 2.4‐fold (from 0.34 to 0.79 g cm^−3^), SLA varied 2.4‐fold (from 8.5 to 20.4 m^2^ kg^−1^), and leaf size varied 21‐fold (from 29 to 614 cm^2^) (Table 1). Traits showed a median 2.6‐fold variation (range 1.4–21.0) and had a median coefficient of variation of 28% (range 12–114%).

**Table 1:** Overview of traits included, the type of trait, trait abbreviation, units, median, 5^th^ percentile, 95^th^ percentile, range (5–95^th^ percentile) and coefficient of variation (CV) of the trait values of rainforest saplings (*n *=* *1007–1309)

Trait category	Abbreviation	Trait name	Units	Median	Percentiles			CV (%)
					5^th^	95^th^	Range 5^th^–95^th^	
Anatomy	BarkProp	Bark proportion	–	0.35	0.17	0.64	0.47	37
	PithProp	Pith proportion	–	0.10	0.02	0.32	0.30	77
	XylProp	Xylem proportion	–	0.52	0.26	0.73	0.47	28
Tissue toughness	BarkD	Bark density	g cm^−3^	0.41	0.23	0.60	0.37	28
	BarkDMC	Bark dry matter content	mg g^−1^	396.48	242.75	547.09	304.34	23
	BranchD	Branch density	g cm^−3^	0.50	0.31	0.69	0.38	23
	FP	Force to punch	N cm^−1^	3.57	2.20	5.81	3.61	29
	FPs	Specific force to punch	N cm^−2^	194.70	103.32	309.44	206.12	32
	LD	Leaf density	g cm^−3^	0.41	0.25	0.56	0.31	23
	LDMC	Leaf dry matter content	mg g^−1^	450.16	289.01	549.38	260.38	18
	PDMC	Petiole dry matter content	mg g^−1^	372.51	204.39	517.04	312.65	27
	WD	Wood density	g cm^−3^	0.57	0.34	0.79	0.46	25
	WDMC	Wood dry matter content	mg g^−1^	532.58	365.55	653.61	288.06	17
Branch size	BarkT	Bark thickness	mm	0.05	0.02	0.12	0.11	63
	BLA_BL_	Branch leaf area per branch length	cm^2^	33.08	11.45	126.62	115.16	99
	BranchA	Branch area	cm^2^	0.17	0.06	0.57	0.51	80
	SBL	Specific branch length	cm g^−1^	12.65	4.01	31.36	27.35	64
Leaf size	LN_BL_	Leaf number per branch length	cm^−1^	0.30	0.12	0.86	0.74	74
	LS	Leaf size	cm^2^	112.76	29.20	614.30	585.10	114
	LT	Leaf thickness	mm	0.19	0.13	0.29	0.16	26
Leaf display	Chl	Chl content	μg cm^−2^	67.84	47.11	94.70	47.59	21
	LAXA	Leaf area per xylem area	cm^2^ cm^−2^	1.47	0.52	3.31	2.78	66
	LAR	Leaf area ratio	cm^2^ g^−1^	104.72	67.01	160.19	93.17	29
	LMF	Leaf mass fraction	g g^−1^	0.81	0.62	0.90	0.28	12
	SLA	Specific leaf area	cm^2^ g^−1^	13.36	8.49	20.35	11.86	28
Whole plant	BA3	Basal area 3^rd^ census	cm^2^	3.80	1.13	15.90	14.77	83
	BA4	Basal area 4^th^ census	cm^2^	3.30	0.95	15.90	14.95	126
	GRBA	Growth rate basal area	cm^2^ yr^−1^	0.07	−0.14	0.66	0.80	228
	GRD	Growth rate diameter	mm yr^−1^	0.17	−0.33	1.33	1.67	193
	HD	Height : diameter ratio	m cm^−1^	1.79	1.04	2.82	1.78	35

The traits are grouped into different trait categories.

**Figure 1:**
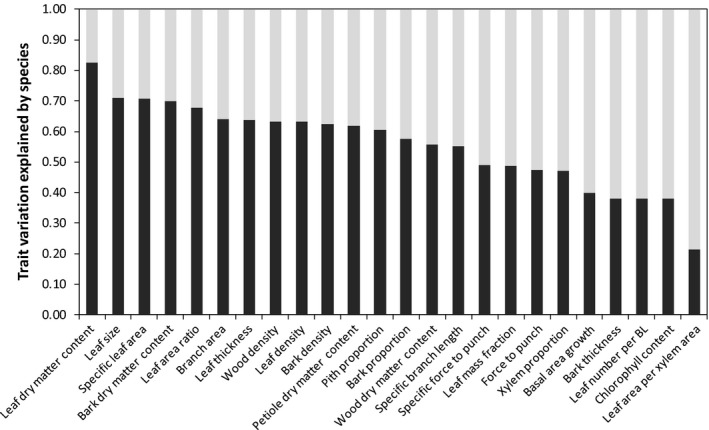
Variance in trait values explained by interspecific variation (black) and intraspecific variation and measurement error (gray). Stacked bars are shown for functional traits and basal area growth. The traits are ranked according to the explained variance by species. Variance calculations were made using all species (*n *=* *47) with at least five individuals (*n *=* *5–40 individuals per species; median, 7 per species).

For the 47 species with sufficient individuals, ANOVA analysis indicated that species explained, on average, 56% of the trait variation, ranging from 22% for leaf area to xylem area ratio to 83% for leaf dry matter content (Fig. 1). The remainder of the trait variation was due to intraspecific variation and measurement error.

### Trait associations and trait trade‐offs

**Figure 2:**
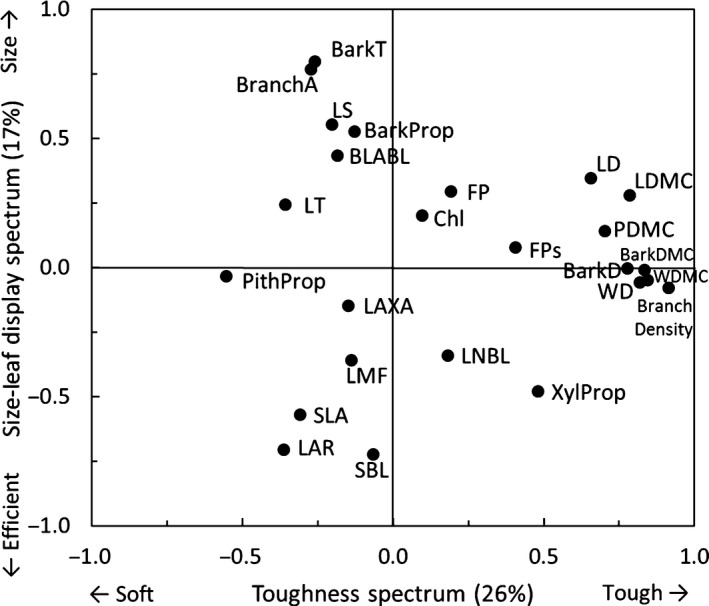
Principal components analysis (PCA) of multivariate trait associations across 920 tropical tree saplings with complete data. The first two PCA axes and the loadings of 25 traits are shown. For trait abbreviations, see Table 1.

To evaluate trait associations and plant strategies, we used a PCA of the 25 traits of the individuals (Fig. 2). The first two PCA axes explained 43% of the variation and showed two spectra of trait variation. The first PCA axis shows saplings with a large proportion of pith at the left, to saplings with tough leaf‐wood and bark tissues (i.e. dry matter content and density) at the right. Hence, this axis represents a toughness spectrum that runs from soft to tough tissues. The second PCA axis shows saplings with large leaves and branches that attain a large leaf area per branch length at the top to saplings with large SLA and LAR at the bottom. Hence, this size–leaf display spectrum runs from large organs at the top to high biomass efficiency for leaf display at the bottom.

**Figure 3:**
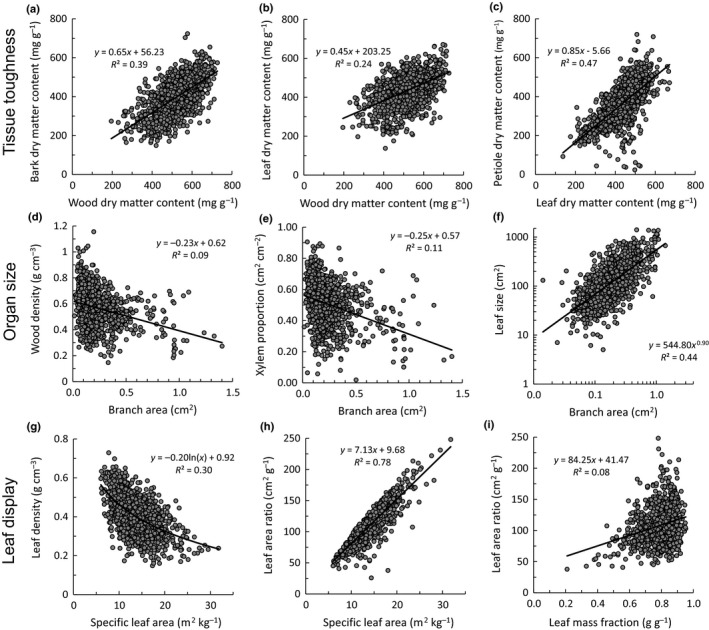
Bivariate relationships between key traits measured at the individual level, across 1300 tropical tree saplings, related to tissue toughness (top panels), organ size (middle panels), and efficiency of leaf display (bottom panels). (a) Bark dry matter content (BMDC) vs wood dry matter content (WDMC), (b) leaf dry matter content (LDMC) vs WDMC, (c) petiole dry matter content (PDMC) vs LDMC, (d) wood density (WD) vs branch cross‐sectional area (Branch A), (e) xylem proportion (XylProp) vs Branch A, (f) individual leaf size (LS) vs Branch A, (g) leaf density (LD) vs specific leaf area (SLA), (h) leaf area ratio (LAR) vs SLA, (i) leaf area ratio (LAR) vs leaf mass fraction (LMF). Regression lines, regression equations, and coefficients of determination are shown.

**Figure 4:**
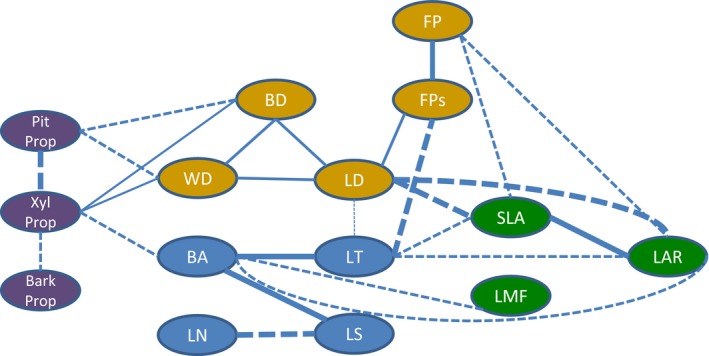
Trait correlation network for saplings of rainforest tree species (*n *=* *1065–1284), showing the main traits and their synergies (positive correlations, continuous lines) and trade‐offs (negative correlations, broken lines). The strength of the trait correlation is indicated by the thickness of the line. Only trait correlations with a Pearson *r *>* *0.3 are shown. Traits are related to branch anatomy (purple ovals; PitProp, pith proportion; XylProp, xylem proportion; Bark Prop, bark proportion), leaf and branch sizes (blue ovals; BA, branch cross‐sectional area; LS, leaf size; LN, leaf number; LT, leaf thickness), tissue toughness (brown ovals; WD, wood density; BD, bark density; LD, leaf density; FP, force to punch; FPs, specific FP), and leaf display (green ovals; LMF, leaf mass fraction; SLA, specific leaf area; LAR, leaf area ratio).

We further explored these trait spectra by looking at pairwise trait associations that were related to spectra in tissue toughness (Fig. 3a–c), organ size (Fig. 3d–f), and leaf display (Fig 3g–i). For the *tissue toughness spectrum*, the dry matter content of the branch tissues (wood, bark) and leaf tissues (leaf lamina, petiole) were strongly positively correlated amongst themselves (Fig. 3a,c), and with each other; that is, individuals with tough branches also had tough leaves (Fig. 3b). In terms of *size*, individuals with soft branch tissue (i.e. low WD) and a low proportion of xylem tended to make thick branches (Fig. 3d,e), and thick branches were, in turn, associated with large leaves (Fig. 3f), and hence a large leaf area per branch length. Leaf size was not related to tissue quality (leaf density) or tissue quantity (leaf thickness). There was a clear trade‐off between the size and the number of leaves that can be packed in a branch (LN_BL_, Spearman *r *=* *−0.57, *P *<* *0.001, Fig. 2), which had consequences for leaf display; a multiple regression indicated that the total branch leaf area per branch length (BLA_BL_) was more driven by variation in leaf size (standardized regression coefficient *β*_std_ = 0.85, *P *<* *0.001) than by leaf number (*β*_std_ = 0.36, *P *<* *0.001), (*r* ^2^ = 0.64, *n *=* *1256). SLA plays a pivotal role in *leaf display*. A multiple regression showed that variation in SLA was equally determined by leaf density (*β*_std_ = −0.87, *P *<* *0.001, Fig. 3g) and leaf thickness (*β*_std_ = −0.81, *P *<* *0.001, *r* ^2^ = 0.64, *n *=* *1283). LAR can be analyzed as the product of SLA and LMF. A multiple regression indicated that LAR is much more driven by SLA (*β*_std_ = 0.98, *P *<* *0.001, Fig. 3h) than by LMF (*β*_std_ = 0.42, *P *<* *0.001, Fig. 3i) (*r* ^2^ = 0.99, *n *=* *1104). The high SLA, though, comes at the expense of a reduced FP (Pearson's *r *=* *−0.37, *P *<* *0.001, Fig. 2). For pairwise trait correlations, see Supporting Information Notes S1; and for the trait correlation network, see Fig. 4.

### Effects of traits and environment on plant growth

To evaluate the effects of environment, size, and traits on basal area growth rate, we fitted a series of models (Table 2). The best growth model included all predictor variables and explained 28% of the variation in individual growth rate. This model included plant height, environment (light and height above the nearest drainage and soil base cations), and all traits, although only four of the traits were significant. Plant height was a better predictor than plant basal area and had the strongest impact on growth, with a standardized effect size *β*_std_ of 0.42–0.48, depending on the model. Light (*β*_std_ = 0.14–0.17) and soil cations (*β*_std_ = 0.13–0.15) had significantly positive effects on growth.

**Table 2:** Overview of eight model comparisons on how environment (light, height above the nearest drainage (HAND), base cations (CMK)), plant size (basal area, height), and leaf and stem traits affect basal area growth rate of tropical tree saplings (*n *=* *580–719)

Model	AIC	*R* ^2^ fixed	*R* ^2^ random	Height	Light	CMK	SLA	FP	WD	LS	Light × LS
Height + all traits + environment	1544	0.24	0.04	0.42	0.14	ns	−0.09	−0.11	−0.08	−0.13	–
Height + best traits	1551	0.21	0.05	0.43	–	–	−0.10	−0.11	−0.09	−0.14	–
Height + all traits	1551	0.21	0.05	0.43	–	–	−0.10	−0.11	−0.09	−0.14	–
Height + best traits + best environment	1765	0.25	0.04	0.42	0.15	0.13	−0.09	−0.07	−0.09	−0.10	–
Height + best traits × Light	1766	0.23	0.05	0.42	0.14	–	−0.08	−0.08	−0.09	−0.09	0.09
Height + best traits × HAND	1769	0.22	0.06	0.42	0.14	–	−0.09	−0.08	−0.09	−0.10	–
Height + environment	1862	0.23	0.05	0.42	0.16	0.15	–	–	–	–	–
Height	1879	0.18	0.08	0.43	–	–	–	–	–	–	–
Height + basal area	1879	0.18	0.09	0.48	–	–	–	–	–	–	–

The models are ranked based on Akaike's information criterion (AIC). The amount of variation explained by the predictor variables (*R* ^2^ fixed) and the plots (*R* ^2^ random) and the effect sizes of the significant predictors are shown. Predictor variables include specific leaf area (SLA), force to punch (FP), wood density (WD), and log_10_‐tranformed individual leaf size (LS) and the interaction between light and LS. The ‘all traits’ models include additional traits that were tested (i.e. log_10_[branch area], pith proportion) but were not significant, and hence are not shown. A dash indicates that variables were not included in the model; ns, not significant.

The growth models that included functional traits performed better than those including only environment, but the amount of additional variation explained by traits was very low (*c*. 3%, by comparing the *r* ^2^ of the model with height with the model with height and all traits). There was no interaction between traits and topography (i.e. height above the nearest drainage), which indicates that the effects of traits on growth are homogeneous along the hydro‐edaphic gradient. All significant traits had similar effect sizes; growth declined with WD (*β*_std_ = −0.08 to −0.09), SLA (*β*_std_ = −0.08 to −0.10), FP (*β*_std_ = −0.08 to −0.11), and leaf size (*β*_std_ = −0.09 to −0.14).

**Figure 5:**
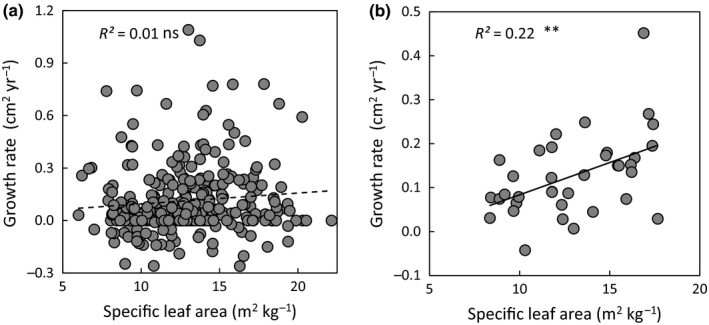
Relationship between specific leaf area and growth rate in basal area using (a) an individual‐based approach (that uses the individually measured traits and growth rates, *n *=* *370 saplings) and (b) a species‐based approach (that uses mean trait values per species, *n *=* *36 species). ns, *P* > 0.05; **, *P* < 0.01.

For a subset of 36 species we checked whether the individual‐based approach and species‐based approach resulted in different relationships between traits and growth rates. For the individual‐based approach, growth was only correlated with tree height, and not with traits, whereas for the species‐based approach the growth rates were significantly positively correlated with SLA (Fig. 5) and LAR, and negatively correlated with leaf thickness (Notes S2).

## Discussion

We screened traits and growth performance of > 1300 individuals in an Amazonian tropical understory tree community and asked how much intraspecific variation there is in trait values, what plant strategies can be distinguished, and whether these traits are indeed functional and predict individual performance. We found that, on average, 44% of the trait variation observed at the community level occurs within species, indicating a strong potential for acclimation to local environmental conditions. Individuals showed two main trait spectra: one related to tissue toughness and another related to organ size vs biomass efficiency of leaf display. Traits measured at the individual level were surprisingly poor predictors of individual growth, whereas traits measured at the species level were reasonable predictors of growth. Here, we discuss the implications of these findings for the ecology and functioning of this tropical community, and for the functional trait approach.

### Large intraspecific trait variation

We first asked how much trait variation is observed in this understory tree community, and how much trait variation is found within and across species. Trait variation was substantial, varying on average by 2.6‐fold across trees (Table 1). For the most abundant species, 44% of the trait variation was found within species (Fig. 1), indicating a strong *potential* for acclimation to current or novel environmental conditions, although it should be noted that measurement errors also contributed to ‘apparent’ within‐species variation, and not all trait variation is due to environmental acclimation. Many comparative trait studies had different objectives when they compared a large number of species within a community, and either ignored (e.g. Reich *et al*., 1999) or underestimated intraspecific trait variation by sampling species under similar environmental conditions (e.g. Rozendaal *et al*., 2006). Many studies compare species under ‘optimal’ growth conditions (i.e. by selecting mature leaves in high resource conditions) so that species can fully express their trait potential (e.g. Poorter & Bongers, 2006), but this leads to an underestimation of the amount of intraspecific variation. Our study has the advantage that it provides a true estimate of trait variation of the species, as many saplings were measured per plot and the plots were systematically distributed across a large landscape, thus sampling most of the environmental variation. A recent meta‐analysis also shows that intraspecific trait variation can be substantial, accounting for 25% of the total trait variation within communities (Siefert *et al*., 2015). See Notes S3 for a discussion on why some traits are more plastic than others.

We hypothesized that least trait variation would be found for tissue quality, because it is phylogenetically more conserved, and indeed found that leaf dry matter content (and to a lesser extent bark dry matter content and bark wood and leaf density) showed least variation within species (Fig 1). We also hypothesized that traits that vary with plant size (leaf area) and adjust to variable light conditions (SLA) would show most variation within species, but in fact found that they were amongst the three least variable traits.

### Trait trade‐offs and plant strategies

Second, we asked how traits are associated and what trade‐offs and plant strategies can be distinguished? We hypothesized that, across individuals, the same leaf and stem economics spectra would be found as reported in the literature across species. We found that leaf and stem traits are closely coordinated. A PCA of all traits showed two axes of trait variation; one axis related to tissue toughness (first PCA axis in Fig. 2) and one axis related to organ size (second PCA axis, top) vs efficiency of leaf display (bottom). These trait spectra are also reflected in the trait correlation network (Fig. 4).

#### Tissue toughness spectrum

The first strategy spectrum is one of tissue toughness; the toughness (i.e. dry matter content and density) of xylem, bark, leaves and petioles was strongly positively correlated (Fig. 3a–c, but see Fortunel *et al*., 2012). Tough tissues are created when most of the tissue volume is filled up by solid and heavy material, such as small cells, thick cell walls, few and small intercellular spaces, and xylem vessels and fibres with small lumen area (Villar *et al*., 2013; Ziemińska *et al*., 2013). Such tough tissues enhance tissue resistance to pathogens, herbivores (Kitajima & Poorter, 2010), and physical damage by wind, rain, trampling and falling debris. As a result, these tough tissues enhance the longevity of leaves, stems, and plants (Loehle, 1988; Sterck *et al*., 2006a; Onoda *et al*., 2011). A tough strategy is especially important in resource‐limited environments, where there is a premium on enhancing carbon and nutrient residence times in plants. For example, leaf dry matter content has been identified as one of the main underlying traits that drive SLA variation, nutrient conservation and plant distribution in temperate herbaceous plants (Hodgson *et al*., 2011). As the Ducke forest occurs on extremely nutrient‐poor soils, this may explain why there is such a strong coordination of toughness traits in our sapling community.

#### Spectrum of size vs efficiency of leaf display

The second strategy axis of variation reflects a trade‐off between size (i.e. the leaf and branch size spectrum, *sensu* Westoby & Wright, 2003) vs biomass efficiency for leaf display. The density of plant tissues has a fundamental impact on branch size and shape. Plants with a low branchwood density need to produce thick branches with wide branchwood area to be biomechanically stable (Fig. 3d) (Sterck *et al*., 2006b; Anten & Schieving, 2010). These wide branches can provide sufficient biomechanical and hydraulic support to sustain large leaves (Fig. 3f). A large leaf area gives rise, in turn, to a large total leaf area per unit branch length (Fig. 2, cf. Falster & Westoby, 2003). These wide and soft branches have relatively little xylem (Fig. 3e), but instead a large proportion of their cross‐sectional area is pith and bark (Fig. 2). A large pith may allow the plant to cut back on branch construction costs, as it consists of parenchyma tissue filled with water or air. Such a ‘hollow tube’ construction is relatively light, and biomechanically very strong for the biomass invested, as all the solid material is displaced to the outer part of the branch. The thick bark protects the soft wood against herbivores and pathogens, provides mechanical strength (Niklas, 1999), and allows the transport of a large amount of sugars. Hence, species with large leaves and branches are able to rapidly occupy a large volume, capture a lot of light, and grow fast. At the opposite side of this size spectrum are individuals with fine and dense wooded twigs that support a large number of small leaves (Fig. 2). These fine twigs also have a large proportion of their cross‐sectional area in xylem, probably to be able to transport sufficient water to the leaves, despite having a narrow twig. This syndrome of soft tissues with wide stems and of large appendices (thick branches, thick bark, and large leaves, flowers, and fruits) is caused by biomechanical and hydraulic coordination, and is also known as Corner's rules (Corner, 1949; Cornelissen, 1999). The size spectrum is probably one of the most widespread and obvious axis of variation in the plant kingdom, and it can have a large impact on plant functioning (Díaz *et al*., 2016). The other side of this spectrum (i.e. the bottom of the PCA) is related to biomass efficiency for leaf display and light capture. It is characterized by saplings that make a large proportional biomass investment in leaves (i.e. they have a high LMF). These leaves have a low leaf density, giving rise to a large SLA (Figs 3g, 4). The high biomass investment in leaves and an efficient leaf display lead to a relatively large leaf area per unit branch mass (LAR) (Figs 3h,i, 4). These branches are not only efficient in leaf display, but also forage efficiently for light, as they have a large branch length per unit branch mass (SBL, Fig. 2). At the whole‐plant level, a large LAR is closely associated with a fast relative growth rate (Poorter & Garnier, 1999). Hence, efficiency of leaf display is most closely related to the leaf economics spectrum and an acquisitive resource use strategy. It should be noted that organ size and leaf display are not totally opposite to each other on the second PCA axis, and instead there seems to be a triangular trade‐off between tissue toughness, size, and leaf display.

### Do plant traits predict individual performance?

Our third question concerned to what extent plant performance is shaped by the environment, plant size, and traits. We hypothesized that plant growth would increase with resource availability (light, water, soil nutrients) and plant size, and indeed found that all but water (i.e. HAND) had strong positive effects on growth. Light was the best environmental predictor, not only because it is a strongly limiting resource, but also because it was measured at the individual level, and hence better linked to individual performance than the other environmental variables that were measured at the plot level. Of the size variables, plant height was a better predictor of growth than stem basal area (Table 2), most likely because height not only reflects plant size (and hence a large photosynthetic leaf area) but it also indicates plant access to diffuse light in the vertical light gradient in the forest canopy. Height and direct light (as indicated by our canopy openness index) were not significantly correlated.

The current paradigm is that ‘fast’ trait values (i.e. high SLA) should lead to fast growth, not only under optimal conditions, but also under suboptimal conditions (Reich, 2014). Many tropical studies indeed find that leaf and plant traits that enhance light capture, such as SLA, crown area, and plant height, can enhance the growth of saplings and larger trees, whereas tissue traits, such as high WD, enhance plant defense and survival but come at the expense of a reduced diameter growth at the species level (Poorter *et al*., 2008; Wright *et al*., 2010; Rüger *et al*., 2012; Iida *et al*., 2014a,b; Fortunel *et al*., 2016). Because the Ducke forest is strongly light and nutrient limited, we hypothesized instead that leaf trait values that enhance leaf lifespan (i.e. high leaf density and FP, and low SLA) would enhance the residence time of carbon and nutrients in the plant, and also lead to a larger leaf area accumulation, light capture, and growth in the shaded understory (cf. Lusk, 2002). We indeed found that low SLA had a positive effect on growth (Table 2). A similar pattern emerges from community‐level studies, which show that under harsh environmental conditions, such as a phosphorus‐poor forest in Guyana (van der Sande *et al*., 2018) or a dry forest in Brazil (Prado‐Junior *et al*., 2016), slow community‐weighted mean trait values (i.e. low SLA) lead to fast community growth. Low SLA leaves do also have a high FP, but surprisingly FP had a negative effect on growth, for which we do not have a clear explanation. Traits associated with water conductance (i.e. high xylem proportion) had a positive effect on growth, as water transport is closely related to gas exchange and carbon gain (Santiago *et al*., 2004). Finally, high WD reduced the stem diameter growth, as high WD implies large volumetric stem construction costs, which is in line with many other studies.

### Why are traits such poor predictors of growth rates?

Although traits did have significant effects on individual growth performance, they explained very little of the growth variation (3%, Table 2). Several ecological, methodological and conceptual reasons can be put forward to explain the weak observed relationship between traits and growth rates.

#### Methodological issues

From a methodological point of view, it could be that we have measured growth imprecisely. In this strongly resource‐limited environment, growth proceeds at slow rate (median diameter growth was only 0.17 mm yr^−1^). Therefore, it is difficult to measure diameter increment accurately. More importantly, stem diameter growth may be a poor descriptor of whole‐plant growth, as trees may rather invest their carbon in height or leaf growth to enhance light capture, in carbon storage to enhance survival in the deep shade, or in root growth to enhance nutrient capture in these very poor soils. It is not likely that these are the main reasons for the weak trait–growth relationships observed, as other field studies found stronger relationships while using similar methods.

#### Ecological issues

From an ecological point of view, it can be that this is just an extremely ‘slow forest’, both trait‐ and growth‐wise. To find relationships between traits and growth rates there should be sufficient variation, both on the *x*‐axis and the *y*‐axis. This Amazonian forest occurs on highly weathered soils that are extremely phosphorus poor (Quesada *et al*., 2012). This may lead to convergence towards conservative trait values, little variation in traits (for a comparison with other forests, see Notes S4), and little variation in growth (e.g. in Ducke DBH growth of *individuals* ranged from −0.33 to +1.33 mm yr^−1^, compared with a range in median species growth of 0–14.8 mm yr^−1^ for similar‐sized trees in a wet forest on more fertile soils in Panama (Welden *et al*., 1991). As a result, there is very little relationship between traits and growth in Ducke.

As nutrients are difficult to acquire, there is a premium on nutrient conservation. Trees from poor Amazonian soils are characterized by extremely dense wood (Muller‐Landau, 2004; ter Steege *et al*., 2006) and tough leaves (Notes S4). The combination of low light and low nutrients may not only lead to slow growth rates, but it may lead to strong trait convergence of the understory community, in which there is a premium on investment in defenses against herbivores (Fine *et al*., 2004) and pathogens (Mangan *et al*., 2010). Hence, in this resource‐poor and slow forest, survival may be more important than growth, and trait values might therefore be better predictors of survival than of growth.

Conservative trait values also have cascading effects on ecosystem functioning and community dynamics. Dense and stiff wood allows trees to produce extremely tall and slender stems, with narrow and deep crowns that cast a deep shade (cf. King *et al*., 2006; Poorter *et al*., 2006). Dense wood is also associated with long lifespans and low tree mortality, resulting in the creation of few canopy gaps (Baker *et al*., 2004); and those gaps are small because, once a tree falls, the narrow crown takes only a few trees down. As a result, there is not only very little light, but also very little light *variation* in the lowest forest layer. This low light (variation) has two consequences for trait–rate relationships. First, trait–growth relationships tend to be stronger under high light, when species can express their full growth potential and maximum growth rates, and tend to weaken under low light, when shade tolerance and persistence become more important (Kitajima & Poorter, 2008; Wright *et al*., 2010; Kunstler *et al*., 2016). Second, because of the few canopy gaps, the Ducke forest contains only a small proportion of pioneer species, which are neither very abundant nor very light demanding. And it is especially very light‐demanding pioneers that tend to have extremely fast traits and fast growth rates, and that have a strong impact on the overall trait–rate relationships.

#### Conceptual issues

From a conceptual point of view, individual‐based and species‐based approaches of trait–growth rate relationships tend to ask different questions, and therefore use different methodologies, and may therefore yield different results. First, the aim of most interspecific studies is to understand how species differ in their performance and why. They tend, therefore, to compare species in a more standardized way under *‘optimal’ growth conditions* (Pérez‐Harguindeguy *et al*., 2013) and they tend to focus more on *potential growth rate*. Such measurement under high light conditions makes the link with potential growth rate stronger, and using standardized conditions reduces the scatter in the trait values. Second, in most of these studies, both the trait and growth values are subsequently averaged out to obtain an *average growth rate* at the species level (but see Iida *et al*., 2014b). Where individual traits and growth may be highly variable in response to stochastic local environment conditions (Fig. 5a), the species‐based approach averages these differences out and shows, therefore, better links between average traits and growth rates (Fig. 5b). Third, by using species rather than individuals as replicates, each species weights equally in the comparisons. This reduces the weight of shade‐tolerant species that have numerous individuals and increases the weight of light‐demanding species that have fewer individuals and more extreme traits and growth rates that can leverage the relationship. Perhaps for these three reasons, trait–rate relationships are stronger in such interspecific studies, as we also found in our forest (Notes S4; Fig. 5b).

By contrast, the aim of most individual‐based approaches is to make a direct link between the traits and the performance of an individual (Violle *et al*., 2007), to take acclimation explicitly into account (Laughlin & Laughlin, 2013), and to get insight into the consequences for community assembly (Violle *et al*., 2012). These studies, therefore, focus more on the *realized growth rate*. Here, ideally the traits of all individuals are measured, as we did in our study. Such an individual approach provides a more realistic view on forest dynamics, because each individual does contribute to forest dynamics. As most individuals in the forest understory belong to shade‐tolerant species with similar convergent traits, and as there is little light‐driven variation, a similar low‐light environment leads to a similar acclimation in traits and growth rates. Moreover, stochastic variation in environmental conditions may decouple traits from growth rates. As a result, there is little relation between traits and growth rates (Fig. 5a), despite the fact that there is a clear gradient in shade tolerance and performance at the species level (Fig. 5b).

## Conclusions

In summary, this screening study has shown that there is substantial trait variation within species. At the individual level, two strategy spectra of variation can be distinguished related to tissue toughness and persistence, and to size vs biomass efficiency of leaf display. These traits and strategy spectra had surprisingly little impact on tree growth, probably because this is a ‘slow’ forest, leading to convergence of both traits and growth rates. However, it can also pinpoint conceptual differences between the species‐based and individual‐based approaches, in which the former focuses on potential and average growth and the latter more on the realized growth. Counterintuitively, the individual‐based trait approach provided less insight on how individuals differ in performance and why, and leads to the question of whether these traits are functional after all. Yet, such an approach may portray a more realistic view on forest dynamics, and may yield, in the end, better insights into community assembly. Future studies using the individual‐based approach should be carried out in more fertile forests to disentangle the relative importance of methodological vs ecological issues on the relationship between functional traits and demographic rates.

## Author contributions

F.R.C.C. and L.P. planned and designed the research, C.V.C. and F.R.C.C. collected the data, L.P. and F.R.C.C. analyzed the data, L.P. wrote the manuscript, and F.R.C.C., C.V.C., J.S. and R.S.O. discussed the results and provided comments.

## Supporting information

Please note: Wiley Blackwell are not responsible for the content or functionality of any Supporting Information supplied by the authors. Any queries (other than missing material) should be directed to the *New Phytologist* Central Office.

**Notes S1** Pearson's correlation table between plant size, light, growth rates and traits across rainforest saplings.
**Notes S2** Relationship between traits and growth rates using an individual‐ and a species‐based approach.
**Notes S3** Discussion why some traits are more plastic than others.
**Notes S4** Comparison between the mean and variation in trait values of trees in tropical dry, moist and wet forest in South America.
