# Supplementary material for: Can traits predict individual growth performance? A test in a hyperdiverse tropical forest
Source: New Phytol. 2018 May 18;219(1):109–21. doi: 10.1111/nph.15206 (PMC6001574; doi:10.1111/nph.15206)
Supplement: Supplementary file 1 — Notes S1 Pearson's correlation table between plant size, light, growth rates and traits across rainforest saplings. Notes S2 Relationship between traits and growth rates using an individual‐ and a species‐based approach. Notes S3 Discussion why some traits are more plastic than others. Notes S4 Comparison between the mean and variation in trait values of trees in tropical dry, moist and wet forest in South America. [file NPH-219-109-s001.pdf]

Supporting information belonging to the article:

Can traits predict individual growth performance? A test in a hyperdiverse tropical forest.
Lourens Poorter, Carolina V. Castilho, Juliana Schietti, Rafael S. Oliveira, Flávia R. C. Costa.
New Phytologist. Acceptance date: 8 March 2018.

Notes S1 Pearson´s correlation table between plant size, light, growth rates and traits across rainforest saplings (median N=1083, range 672-1318). For trait abbreviations see Table 1. Significant correlations (P<0.05) are given in bold.

Category	Trait	Height	LightTre	BA4	BA3	GRBA	GRD	BarkPro	PithProp	XylProp	BarkD	BarkDM	BranchD	FP	FPs	LD	LDMC	PDMC	WD	WDMC	BarkT	BLA_{BL}	BranchA	SBL	LN_{BL}	LS	LT	Chl	LAXA	LAR	LMF	SLA
Whole plant	Height	1.00																														
	LightTree	0.01	1.00																													
	BA4	**0.49**	0.02	1.00																												
	BA3	**0.67**	**0.10**	**0.64**	1.00																											
	GRBA	**0.28**	**0.15**	**0.43**	0.06	1.00																										
	GRD	**0.19**	**0.14**	**0.29**	**-0.07**	**0.95**	1.00																									
Anatomy	BarkProp	-0.03	0.01	-0.03	0.03	**-0.09**	**-0.09**	1.00																								
	PithProp	-0.02	0.07	-0.05	0.01	0.07	**0.09**	**-0.27**	1.00																							
	XylProp	0.04	**-0.06**	**0.06**	-0.03	0.04	0.03	**-0.76**	**-0.41**	1.00																						
Tissue toughness	BarkD	**0.10**	-0.02	0.02	-0.05	-0.03	-0.03	**-0.11**	**-0.32**	**0.32**	1.00																					
	BarkDMC	**0.08**	-0.05	0.03	-0.04	-0.02	-0.03	**-0.12**	**-0.33**	**0.33**	**0.83**	1.00																				
	BranchD	**0.06**	**-0.07**	0.00	-0.07	-0.05	-0.05	**-0.19**	**-0.45**	**0.48**	**0.77**	**0.78**	1.00																			
	FP	-0.01	0.03	0.04	-0.01	-0.04	-0.02	**0.11**	-0.03	**-0.08**	**0.06**	**0.06**	0.06	1.00																		
	FPs	0.00	0.01	**0.06**	-0.02	-0.06	-0.05	0.01	**-0.15**	**0.09**	**0.20**	**0.22**	**0.18**	**0.69**	1.00																	
	LD	**0.09**	0.04	**0.09**	0.01	0.04	0.03	**0.07**	**-0.23**	**0.09**	**0.36**	**0.38**	**0.38**	**0.19**	**0.44**	1.00																
	LDMC	0.05	0.02	0.05	-0.03	-0.01	-0.01	0.05	**-0.34**	**0.18**	**0.58**	**0.63**	**0.57**	**0.15**	**0.33**	**0.67**	1.00															
	PDMC	0.02	0.00	0.03	-0.01	-0.03	-0.04	**0.06**	**-0.35**	**0.18**	**0.53**	**0.58**	**0.55**	**0.10**	**0.30**	**0.45**	**0.68**	1.00														
	WD	-0.01	**-0.09**	-0.02	**-0.08**	**-0.10**	**-0.10**	**-0.07**	**-0.47**	**0.38**	**0.47**	**0.59**	**0.86**	**0.07**	**0.16**	**0.35**	**0.46**	**0.47**	1.00													
	WDMC	0.01	**-0.08**	-0.02	-0.06	-0.05	-0.06	**-0.06**	**-0.57**	**0.44**	**0.55**	**0.62**	**0.83**	0.05	**0.17**	**0.36**	**0.49**	**0.49**	**0.86**	1.00												
Branch size	BarkT	0.03	0.05	0.02	**0.10**	-0.05	-0.06	**0.79**	**-0.07**	**-0.70**	**-0.22**	**-0.23**	**-0.31**	**0.14**	-0.01	0.06	-0.02	-0.05	**-0.22**	**-0.24**	1.00											
	BLA_{BL}	**0.11**	0.02	**0.07**	**0.10**	0.00	-0.01	0.01	**0.15**	**-0.11**	**-0.13**	**-0.13**	**-0.16**	0.03	0.01	0.00	-0.01	-0.02	**-0.18**	**-0.19**	**0.33**	1.00										
	BranchA	**0.09**	0.06	**0.07**	**0.14**	0.03	0.01	**0.20**	**0.20**	**-0.32**	**-0.22**	**-0.21**	**-0.29**	**0.09**	-0.03	0.03	-0.05	**-0.12**	**-0.29**	**-0.31**	**0.71**	**0.61**	1.00									
	SBL	**-0.09**	-0.03	**-0.08**	-0.05	-0.02	0.00	**-0.19**	0.03	**0.16**	**-0.07**	**-0.07**	-0.05	**-0.16**	-0.03	**-0.21**	**-0.14**	-0.05	-0.03	-0.05	**-0.48**	**-0.35**	**-0.59**	1.00								
Leaf size	LN_{BL}	0.02	-0.01	**0.06**	0.05	0.04	0.02	**-0.09**	**-0.17**	**0.20**	0.05	**0.08**	**0.16**	**-0.10**	0.00	-0.03	-0.01	**0.12**	**0.17**	**0.15**	**-0.17**	**0.07**	**-0.19**	**0.20**	1.00							
	LS	**0.09**	0.01	0.02	0.02	0.01	0.02	0.01	**0.18**	**-0.13**	**-0.07**	**-0.08**	**-0.18**	0.04	0.05	0.02	0.04	0.02	**-0.24**	**-0.22**	**0.36**	**0.73**	**0.67**	**-0.42**	**-0.33**	1.00						
	LT	-0.02	0.03	-0.03	0.02	0.05	0.04	**0.09**	**0.19**	**-0.22**	**-0.21**	**-0.23**	**-0.19**	**0.22**	**-0.50**	**-0.41**	**-0.27**	**-0.28**	**-0.15**	**-0.18**	**0.17**	0.02	**0.16**	**-0.16**	**-0.08**	-0.01	1.00					
Leaf display	Chl	0.04	-0.01	0.02	0.05	**0.09**	**0.09**	0.04	**-0.06**	0.00	0.00	-0.01	0.03	**0.07**	-0.04	**0.15**	**0.11**	0.04	**0.06**	**0.01**	**0.07**	-0.05	0.05	**-0.07**	**-0.12**	-0.01	**0.14**	1.00				
	LAXA	-0.03	0.00	-0.04	0.01	-0.01	0.02	**0.09**	**0.22**	**-0.23**	-0.05	-0.04	**-0.06**	0.06	**0.07**	**-0.10**	-0.05	0.02	-0.04	**-0.10**	-0.02	**0.31**	**-0.14**	**0.16**	**0.16**	**0.12**	-0.02	**-0.06**	1.00			
	LAR	**-0.08**	-0.04	-0.05	-0.01	-0.04	-0.02	**-0.19**	**0.11**	**0.10**	**-0.22**	**-0.21**	**-0.24**	**-0.30**	0.02	**-0.53**	**-0.44**	**-0.24**	**-0.23**	**-0.23**	**-0.31**	-0.01	**-0.31**	**0.48**	**0.24**	**-0.11**	**-0.35**	**-0.25**	**0.32**	1.00		
	LMF	-0.04	0.00	-0.04	-0.02	0.03	0.04	**-0.06**	**0.09**	0.00	**-0.18**	**-0.17**	**-0.12**	**0.13**	-0.06	-0.02	**-0.12**	**-0.17**	-0.05	**-0.08**	**-0.26**	0.00	**-0.39**	**0.35**	**0.29**	**-0.25**	**0.21**	0.05	**0.45**	**0.26**	1.00	
	SLA	**-0.06**	-0.04	-0.05	0.00	**-0.07**	-0.06	**-0.17**	**0.08**	**0.11**	**-0.14**	**-0.13**	**-0.17**	**-0.36**	0.04	**-0.54**	**-0.39**	**-0.17**	**-0.18**	**-0.17**	**-0.22**	-0.02	**-0.16**	**0.33**	**0.13**	-0.02	**-0.46**	**-0.28**	**0.13**	**0.90**	**-0.17**	1.00

Notes S2 Relationship between traits and growth rates using an individual-based approach (that uses traits and growth rates of 370 saplings) and a species-based approach (that uses mean trait values per species for 36 species). Pearson's correlations (*r*) and coefficients of determination (R^2) and shown.

Trait category	Trait	Individual		Species		Difference
		R	R^2	*R*	R^2	R^2ind - R^2sp
Anatomy	BarkProp	-0.052	0.003	-0.305	0.093	-0.090
	PithProp	0.054	0.003	0.155	0.024	-0.021
	XylProp	0.023	0.001	0.203	0.041	-0.041
Tissue toughness	BarkD	0.066	0.004	0.090	0.008	-0.004
	BarkDMC	0.080	0.006	0.105	0.011	-0.005
	BranchD	0.017	0.000	0.033	0.001	-0.001
	FP	-0.019	0.000	-0.202	0.041	-0.040
	FPs	0.034	0.001	0.146	0.021	-0.020
	LD	-0.017	0.000	-0.168	0.028	-0.028
	LDMC	0.045	0.002	-0.015	0.000	0.002
	PDMC	0.037	0.001	0.018	0.000	0.001
	WD	-0.038	0.001	-0.010	0.000	0.001
	WDMC	-0.042	0.002	0.007	0.000	0.002
Branch size	BarkT	-0.007	0.000	-0.275	0.076	-0.076
	BLA_{BL}	-0.017	0.000	-0.017	0.000	0.000
	BranchA	0.059	0.004	-0.106	0.011	-0.008
	SBL	-0.040	0.002	0.063	0.004	-0.002
Leaf size	LN_{BL}	0.047	0.002	-0.067	0.004	-0.002
	LS	-0.018	0.000	0.010	0.000	0.000
	LT	-0.085	0.007	**-0.384**	0.147	-0.140
Leaf display	Chl	-0.027	0.001	-0.056	0.003	-0.002
	LAXA	0.013	0.000	0.266	0.071	-0.070
	LAR	0.069	0.005	**0.387**	0.150	-0.145
	LMF	-0.067	0.005	-0.285	0.081	-0.076
	SLA	0.100	0.010	**0.473**	0.224	-0.214
Whole plant	BA3	0.076	0.006	-0.147	0.022	-0.016
	Height	**0.256**	0.066	-0.082	0.007	0.059
	Light Tree	0.108	0.012	0.146	0.021	-0.010

Significant correlations ($P<0.05$) are shown in bold. The last column indicates the difference in explanatory power between the individual-based approach (R^2 individuals) and the species-based approach (R^2 species). For trait abbreviations see Table 1.

Notes S3. Discussion why some traits are more plastic than others

We hypothesized that traits related to organ size and leaf display would be strongly related to tree sapling size and light, and would vary therefore mostly within species, whereas traits related to tissue quality and toughness would be phylogenetically more conserved and therefore vary also little within species. We indeed found high intraspecific trait variation, ranging from 17-78% across traits. As expected, traits related to tissue toughness (e.g., dry matter content and density) showed the least intraspecific variation, as they tend to be phylogenetically conserved (Chave *et al.*, 2006). Surprisingly, several leaf display traits (SLA and LAR) showed relatively little intraspecific variation, which strongly contrasts with controlled studies, which have shown that leaf display traits change strongly with the light environment, to enhance light capture and carbon gain (Poorter, 1999; Poorter *et al.*, 2009; Sterck *et al.*, 2013). Perhaps in our study intraspecific variation in SLA and LAR was smaller than expected, because they did not respond to our quantitative index of crown exposure. We predicted that size-related traits should be very plastic in response to tree sapling size, but found instead that they show little intraspecific variation. For example, leaf size was the trait that showed the second-lowest variation within species (Fig. 1). Alternatively, intraspecific trait variation is simply the consequence of ontogenetic drift, tissue ageing, and wear and tear, which raises the question whether this large trait variation is functional, and whether it also implies a large trait acclimation.

Notes S4 Comparison between the mean and variation in trait values of trees in tropical dry, moist and wet forest in South America. Mean and variation (coefficient of variation, CV in %) in trait values of branch wood density (WD), leaf dry matter content (LDMC), specific leaf area (SLA) and individual leaf size (LS) are shown.

Trait	Mean			Variation (CV)		
	Dry	Moist	Wet	Dry	Moist	Wet
WD (g cm^{-3})	0.614 c	0.504 a	0.571 b	33	45	23
LDMC (mg g^{-1})	288 a	312 b	438 c	31	20	18
SLA (m^2 kg^{-1})	21.8 c	19.9 b	13.8 a	32	27	28
LS (cm^2)	51 a	73 b	120 c	144	194	114

Data from the wet forest (rainfall 2550 mm y^{-1}) come from Ducke Reserve , Manaus (this study, n=1251-1300 saplings), data from the moist semi-evergreen forest (rainfall 1580 mm y^{-1}) come from La Chonta, Bolivia (Rozendaal *et al.*, 2006) (n=189 trees), and data from the dry deciduous forest (1160 mm y^{-1}) come from INPA, Bolivia (Markesteijn *et al.*, 2007) (n=198 trees). All traits differed significantly amongst forest types (ANOVA, P<0.001 in all cases). Means followed by a different letter are significantly different (Tukey post-hoc test, P<0.05). For the ANOVA, LS has been log_{10}-transformed, but in this table back-transformed values are shown. The CV of LS is based on untransformed values.

In the Bolivian studies trait data have been sampled with a different sampling design, so they are not strictly comparable with Ducke, but they are included here to illustrate the point how these forests might differ. In Bolivia traits have been sampled for shaded trees, but larger trees (between 10-20 cm cbh) compared to Ducke (1-5 cm DBH). In Bolivia, traits were sampled for 37-38 abundant species, differing in shade tolerance, 5 trees per species. Because of this design, not the whole community was sampled, and trait values may potentially show a larger spread because species were deliberately selected that differed in their strategy. To correct for this, we weighted the Bolivian trait values for the tree abundance of these species in 32-48 1-ha plots, where all trees >10 cm dbh were measured (Peña-Claros *et al.* 2012). Weighting was only done, based on the relative stem abundance of those 36 species for which the traits were measured. For Ducke each measured stem weighted as 1.

The difference in sampling design may have affected the results in two ways. Within species, taller trees tend to have more conservative trait values (i.e., higher WD, LDMC, LS and lower SLA), which may make the trait means of the Bolivian forests more similar to that of the conservative forest in Ducke, thus underestimating mean trait differences. Because in the Bolivian forest different strategies were sampled we may have overestimated their trait variation (CV) even when correcting for stem abundances. Because in Bolivia not the whole community and all plants in different micro environmental conditions were measured, we may have underestimated their trait variation (CV).

In terms of average trait values, saplings in the wet and nutrient poor Ducke forest have, compared to the other forests high LDMC and low SLA , which enhance leaf longevity

and nutrient residence time in the plant. Similarly, branchwood density was also relatively high, but higher in the dry deciduous forests where high WD enhances cavitation resistance and drought tolerance. Leaf size was largest in the wet forest of Ducke.

References

Chave J, Muller-Landau HC, Baker TR, Easdale TA, Ter Steege H, Webb CO. 2006. Regional and phylogenetic variation of wood density across 2456 neotropical tree species. *Ecological Applications* **16**: 2356-2367.

Markesteijn L, Poorter L, Bongers F. 2007. Light-dependent leaf trait variation in 43 tropical dry forest tree species. *American Journal of Botany* **94**:515-25.

Peña-Claros M, Poorter L, Alarcón A, Blate G, Choque U, Fredericksen TS, Justiniano MJ, Leaño C, Licona JC, Pariona W, Putz FE. 2012. Soil effects on forest structure and diversity in a moist and a dry tropical forest. *Biotropica* **44**:276-283.

Poorter L. 1999. Growth responses of 15 rain-forest tree species to a light gradient: the relative importance of morphological and physiological traits. *Functional Ecology* **13**: 396-410.

Poorter H, Niinemets U, Poorter L, Wright IJ, Villar R. 2009. Causes and consequences of variation in leaf mass per area (LMA): a meta-analysis. *New Phytologist* **182**: 565-588.

Rozendaal DMA, Hurtado VH, Poorter L. 2006. Plasticity in leaf traits of 38 tropical tree species in response to light; relationships with light demand and adult stature. *Functional Ecology* **20**: 207-216.

Sterck FJ, Duursma RA, Pearcy RW, Valladares F, Cieslak M, Weemstra M. 2013. Plasticity influencing the light compensation point offsets the specialization for light niches across shrub species in a tropical forest understorey. *Journal of Ecology* **101**: 971-980.
